# Impact of parasitic infection with *Ascaris lumbricoides* on pulmonary function tests in asthmatic and non-asthmatic children

**DOI:** 10.1016/j.rmcr.2021.101552

**Published:** 2021-11-10

**Authors:** Suha Haithem Mohammed, Azza Sajid Jabbr, Nawal Khalil Ibrahim

**Affiliations:** a Department of Clinical Laboratory Sciences, College of Pharmacy, University of Basrah, Iraq; b Department of Pharmacology and Toxicology, College of Pharmacy, University of Basrah, Iraq; c Department of Physiology, College of Medicine, University of Basrah, Iraq

**Keywords:** Ascaris lumbriocoides, Asthma, Pulmonary function tests

## Abstract

**Background:**

There is strong evidence for a causal relationship between helminthes infection and allergic disease like asthma due to the similarity in the way they respond. This study aimed to investigate the effects of Ascaris infection on pulmonary function tests (PFTs) to reveal the relationship between ascariasis and asthma in children.

**Patients and methods:**

This a randomized-control study conducted in Basrah City, Iraq, in which four groups of a total of 490 children were enrolled: Group1 included 120 normal children; Group 2 included 135 asthmatic children; Group 3 who were 150 Ascaris infected children and group G4 included 85 asthmatic and Ascaris infected. PFTs, IgE level, differential blood count and parasitic examination were done for all groups.

**Results:**

Both group2 and 4, which included asthmatic children showed a significant decrease in PFT (P > 0.05),while the PFT of parasitic infected group was not affected. There were no significant changes in WBC, eosinophils and IgE between asthmatic and parasitic infected groups.

**Conclusion:**

Ascaris infection could induce the inflammatory immune response in children, but couldn't cause a significant effect on pulmonary function tests in these children; The impairment in PFT was due to asthma disease and not correlated to ascariasis.

## 1 Introduction

Parasitic infection and allergic disease are the major public health problems [1]. There is a strong evidence for a causal relationship between helminthes infection and allergic disease [2]. The way that human body's immune system responds to allergens is very similar to the way it responds to parasitic worms infections [3],that characterized by an increase in serum IgE and the number of eosinophils (eosinophilia) [4].

It has been found that helminthic infections,which are intestinal or soil transmitted including *Ascaris lumbricoid*, hookworm and trichuris were common parasitic infections and widely distributed all over the world. These infections are characterized by high prevalence among children specially who live in rural regions with below the standard of the sanitation and the healthy conditions [5]. Ascariasis is a widespread infection all over the world,therefore it is considered a serious problem to the public health [6,7].

Although the growing researches and reports about the interrelation between parasite infections and allergic response, that obtained from observations in humans [8],this interrelation still uncertain, as well as some evidences suggest that *Ascaris lumbricoides* is a neglected risk factor for asthma [9]. Asthma is a common allergic airway disease is characterized a chronic inflammatory disorders of the airways that results in a variable air flow limitation [10]. Pulmonary function tests(PFT) are noninvasive detectable tests used to show how the lung is functioning by measuring the volumes and capacities of the lung [11]. This study aimed to investigate the effects of Ascaris infection on PFT to reveal the relationship between ascariasis and asthma and to find out if the measurement of PFT may work as a supportive protocol to diagnose the infection of Ascaris in children.

## 2 Material and methods

### 2.1 Patients

This study is a randomized controlled trial, conducted in Basrah City, Iraq. The source of patients was the students of the intermediate schools and individuals attending one of the primary health care centers. The individuals enrolled in the study were divided into four groups as the following: Group1(G1) included 120 normal individuals (64 males,56 females),with mean of age 15.28 ± 9.50 years. Group 2(G2) included 135 asthmatic individuals(74 males,61 females) with age mean 14.39 ± 8.33 years. Group 3 (G3) who were 150 parasitic infected individuals with *Ascaris lumbricoides* (83 males,67 females) with age of mean 13.19 ± 8.97 years and the fourth group (G4)included 85 individuals (48 males,37 females) who were asthmatic and infected with Ascaris at the same time,the mean age was(14.26 ± 9.42) years.

The contributed individuals were apparently healthy and free of any other diseases (rather than asthma concerning G2 and G4), that may impact pulmonary function tests (PFT), complete blood count or total IgE. Moreover several criteria were excluded from the study such as obesity, (the individuals of all groups were with normal BMI), individuals who were chronically ill or on medications for long duration; with history of any major surgery; undergoing any physical conditioning program; history of active sports training at school and suffering from arthritic disorders, skeletal deformities, or any other abnormalities. The characteristics of all participants were obtained by a detailed form of questionnaire. This work was reported in line with the CONSORT statement and approved by a local ethical committee of college of Pharmacy,University of Basrah.

### 2.2 Methods

Pulmonary function tests (PFT) for all individuals of the groups were measured by MicroMedical Spirometer instrument (MIR SpirolabIII Diagnostic Spirometer,Ltd. England). It is suitable for accurate and early diagnosis of respiratory diseases (like COPD and Asthma) and CRPD. The spirometry applied to all participants in a same position. the individual^,^s details(sex,age,weight, hight and race) were all recorded. The participant were instructed to exhale the air forcefully and continuously in the mouthpiece of the instrument. In order to get the best result,the procedure was repeated three times for each individual. FVC function was chosen to record the required parameters and diagnosis, According to the American Thoracic Society (ATS), the normal pulmonary function tests are when the values of FVC and FEV1 normal. Reductions in them or even one refers to pulmonary disorder [12].

Obstructive lung disorders were considered when FEV1/FVC< 70%, restrictive lung disorder is considered when FVC<80% and combined (restrictive and obstructive) lung disease were diagnosed when both (FVC and FEV1/FVC) are reduced [13].

#### 2.2.1 Blood sample collection

Four milliliters of blood was drawn from each individual by antecubital venipuncture approach using disposable syringe. The blood were collected in two tubes. One tube containing EDTA (disodium ethylenediamine tetra-acetate) for measuring hematological indices. Total white blood cell count and differential count (neutrophils, basophils and eosinophils), using the auto hematology analyzer (Ruby, Germany). The other is a gel plain tube to prepare the sera for measuring total IgE. The tubes kept in an icebox till to the measurement time, which was performed on the same day of collection. The laboratory works were done in a private laboratory for analyzes.

#### 2.2.2 Serum total IgE measurement

Total IgE was estimated using ELISA (Enzyme Liked Immunosorbant Assay) kit (Euroimmun-Germany),that gives quantitative measurement in vitro test for IgE of human. The procedure was done by following manufacture instructions. The serum was measured by mean of calibrated curve that obtained by calibration the serum from 1 to 4 that contains different concentrations of IgE (0,10,100,250 IU/ml) [14].

#### 2.2.3 Parasite examination

Fecal specimen was collected from all individual in the study to detect the presence of Ascaris helminthes. Direct microscopic fecal examination (wet mount) was used to detect the presence of the parasite. Formalin (sodium acetate-acetic acid) was used to preserve the content of the specimen. Fecal specimen that contained any parasite other than Ascaris was excluded from the study [15].

### 2.3 Statistical analysis

The data were statistically analyzed using Statistical Package for the Social Sciences (SPSS) Statistical Software for Windows, Version 24.0 IBM (SPSS Inc, IL, USA). They were represented as means value ± standard deviation (SD) and using (Independent student-test and one way a nova to measure the differences between the mean values of the two parameters. Pearson's correlation test was used to get the correlation between the parameters. If the result was p < 0.05 it considered significant.

## 3 Results

This is a randomized controlled study conducted for the period between January and May, 2021. A total of 490 children enrolled in the study and categorized in different groups, as mentioned in materials and methods, showed no significant differences (p < 0.05) in gender ratio, mean of age and BMI(kg/m2), as shown in a Table 1.

**Table 1:** Characteristics of the groups.

Group Parameter	G1 N = 120	G2 N = 135	G3 N = 150	G4 N = 85	P value
Males Females	64 (53.33%) 56 (46.66%)	74(54.8%) 61(45.18%)	83(55.33%) 67(44.66%)	48(56.47%) 37(43.5%)	0570. 0.053
Age	15.28 ± 9.50	14.39 ± 8.33	13.19 ± 8.97	14.26 ± 9.42	0.61
BMI	20.23	19.31	18.54	21.4	0.86
Respiratory diagnosis	Normal	Mild Obstructive	Normal	Mild Obstructive	

**When comparing the pulmonary function tests among the groups, we found significant differences** (p > 0.05) **in FEV1 between G1 and G2** (3.2700 ± 0.38 vs 2.4478 ± 0.36), between G1 and G4 (3.2700 ± 0.38 vs.2.36 ± 0.43) and between G2 and G3 (2.4478 ± 0.36 vs. 3.430 ± 0.53). On the other hand, it was non-significant difference between G1(healthy children) and G3 (Ascaris infected children),(p < 0.05),as shown in Table 2. The same findings were reported regarding both FEV1% and PEF. There was no significant difference in FEV1% between G1 and G3 (85.6502 ± 6.45 vs. 86.1502 ± 7.15), (p < 0.05). While, there were significant differences (p > 0.05) between G1 and G2 (85.6502 ± 6.45 vs. 76.2026 ± 7.05),between G1 and G4(85.6502 ± 6.45 vs. 75.32 ± 6.25) and the important finding was the significant difference between G2(asthmatic children) and G3 (Ascaris infected children) (76.2026 ± 7.05 vs. 86.1502 ± 7.15),(p > 0.05),as seen in Table 2. Morover the table shows same outcome results regarding the PEF parameter:no significant difference between G1 and G3(p < 0.05),while there was a significant difference in between G2 and G3(p > 0.05). On the other hand, the mean value of FVC showed no significant changes among the different groups, (p < 0.05),(Table 2).

**Table 2:** Pulmonary function tests Comparison among the different groups.

Group Parameter	G1	G2	G3	G4	P value Between G 1 & (2,3,4)	P value Between G2&(3,4)	P value Between G3&4
FEV1	3.2700 ± 0.38	2.4478 ± 0.36	3.430 ± 0.53	2.36 ± 0.43	*0.011 0.082 *0.021	*0.01 0.12	*0.031
FVC	3.8146 ± 0.38	3.3425 ± 0.33	3.921 ± 0.46	3.56 ± 0.35	0.52 0.64 0.66	0.93 0.87	0.65
FEV1%	85.6502 ± 6.45	76.2026 ± 7.05	86.1502 ± 7.15	75.32 ± 6.25	*0.021 0.091 *0.015	*0.01 0.41	*0.022
PEF	7.7581 ± 0.82	6.63 ± 0.85	8.21 ± 1.02	5.64 ± 0.72	*0.033 0.091 *0.019	*0.024 0.63	*0.024

* Data are considered significant at p > 0.05.

Analysis of hematological and IgE data revealed that the mean values of WBC were significantly different (p > 0.05) between each two groups in the study, as seen in Table 3. The only non-significant change was between G2(asthmatic children) and G3(Ascaris infected children) (6100 ± 312 vs. 6230 ± 281.3),(p < 0.05). While the only significant change in mean value of neutrophils was between G1 and G2(3.11 ± 1.02 vs. 4.95 ± 1.6), (p > 0.05). No significant changes were found among the four groups in basophils. Table 3 also showed that there were significant differences in eosinophils, (p > 0.05) between each of G1 and G3 (0.3 ± 1.32 vs. 0.73 ± 0.92), between G1 and G4(0.3 ± 1.32 1.03 ± 0.37) and between G2 and G4(0.45 ± 1.12 vs. 1.03 ± 0.37).

**Table 3:** Blood parameters and IgE Comparison among the different groups.

Group Parameter	G1	G2	G3	G4	P value Between G 1 &(2,3,4)	P value Between G2&(3,4 (	P value Between G 3 &4
WBC	4198.4 ± 337.5	6230 ± 281.3	6100 ± 312.6	6630 ± 291.3	<0.001٭٭ <0.001٭٭ <0.001٭٭	0.064 *0.031	*0.016
Neutrophils	3.11 ± 1.02	4.95 ± 1.6	3.96 ± 1.39	4.65 ± 1.8	*0.02 0.046 0.012	0.042 0.091	0.062
Basophils	0.1 ± 1.12	0.21 ± 0.76	0.23 ± 0.33	0.11 ± 0.54	0.096 0. 82 0.17	0.21 0.13	0.43
Eosinophils	0.3 ± 1.32	0.45 ± 1.12	0.73 ± 0.92	1.03 ± 0.37	0.062 *0.012 *0.01	0.072 *0.014	0.45
IgE	95.45 + 90.6	197.25 ± 167.3	212 ± 94.2	231 ± 201.66	*0.022 *0.010 *0.0042	*0.036 *0.015	0.75

* Data are considered significant at p > 0.05.

Data analysis to find the correlation between FEV1% parameter and differential count parameters and IgE level revealed non-significant correlations between FEV1% and each of blood parameters and IgE (p < 0.05),as seen in Table 4.

**Table 4:** The Correlation between FEV1% parameter and differential count parameters and IgE level.

Parameters	r	p
FEV1% &WBC	**- 0.421**	**0.052**
FEV1%& Neutrophils	**0.366**	**0.062**
FEV1%& Basophils	**0.216**	**0.142**
FEV1%& Eosinophils	**- 0.357**	**0.131**
FEV1%& IgE	**- 0.467**	**0.057**

Correlation is significant when p > 0.05 (2-tailed).

Regarding the IgE, we found significant differences between G1 and every other group in the study (p > 0.05), as well as significant differences between G2 and both G3 and G4. The only non-significant change was between G3and G4,(p < 0.05),as seen in Table 3.

## 4 Discussion

Data analysis revealed that all participants of the four groups were non significantly different in gender ratio, mean of age and BMI (kg/m^2^),(p < 0.05) as seen in Table 1. Regarding pulmonary function tests(PFT),the respiratory diagnosis of asthmatic children in the study was mild obstruction. The groups of the study showed some significant variations in pulmonary function tests(FEV1,FEV1% and PEF) as the following: a significant decline(p > 0.05) in these parameters in (G2) and (G4), the two groups that included asthmatic children. On the other hand,G3 revealed non-significant difference in pulmonary function tests compared to G1,the healthy control group (p < 0.05) but a significant difference compared with asthmatic G2, (p > 0.05). The result of our study refers to that Ascaris infection did not affect the PFT. The significant change in the PFT was only due to the effect of asthma, even in case of mild obstruction. Asthma results in a decline in PFT especially FEV1 and FEV1% because of the limitation of airflow that occurs as a result of the allergic inflammatory process which characterized by edema of air passages and bronchoconstriction that lead to increased airway resistance [[16], [17], [18], [19]]. This result was consistent with previous studies [11,18].

Several previous studies have pointed to that ascaris infection might induce the allergic inflammatory processes and allergic airways symptoms such as asthma [[20], [21], [22]]. However these studies did not measure the PFT of the infected children with Ascaris as we did in this study. It should be mentioned infected individual with limited exposure to helminthes could induce allergic manifestation response like asthma disease. The allergic response developed due to passing the larvae of *Ascaris lumbericoides* through the lungs [23]. The helminthes infections may affect the allergic reaction and the inflammatory responses by either stimulation or inhibition the allergic inflammatory processes against the parasites; immunological cross reaction between the allergen and aeroallergen and by effect of allergic inflammatory processes against aeroallergens in the same tissue [[24], [25], [26]].

On the other hand,different findings were reported by other studies. One concluded that parasitic infections with helminthes were correlated to decreased risk of asthma like disease symptoms [27], whereas another study found that the allergic reactions was not correlated to parasitic infections in children [28]. In general it has been found that parasites with limited and restricted infection into the intestinal lumen are less likelihood to result in that strong systemic immune reactions. Moreover impact of helminthic infections in allergy may be related to several factors such as times of exposure to the parasite; severity of the parasitic infections as well as the genetic factors of the infected individual [21]. These factors may explain the non-significant results regarding the FPT reported in this study.

Regarding the hematological parameters and IgE level, we found significant variations (p > 0.05) in each of WBC, eosinophil and IgE level between G1 and each of the other groups, as shown in Table 3. While the difference between G2 and G3 in these parameters was not significant (p < 0.05),in contrast with the significant result related to PFT. This result points to the fact that the impact of Ascaris infection was associated with increased level of WBC, eosinophils and IgE in a similar profile to that change of asthmatic, but this impact was unrelated to PFT. Children immune responses to this parasitic infection resulted in increased level of WBC, eosinophils and total IgE, a result came in agreement with what were pointed by previous studies,an individual immune response to the presences of helminthic parasites led to elevated level of IgE, eosinophilia,mastocytosis and interleukin 4,5 and 13 as well as presence of parasites in the tissue might induce localized Th2 response that characterized by formation of inflammatory infiltrate rich with eosinophils [29,30].

## 5 Conclusion

We concluded that Ascaris infection could induce the immune response in children, represented by significant changes in WBC, eosinophils and IgE,but couldn't exert such significant effect on pulmonary function tests in these children; The PFT of asthmatic children were significantly less than that of Ascaris infected children and the impairment in PFT was due to asthma disease and not correlated to Ascaris infection. The finding could be explained by several factors mentioned earlier Furthermore, some points are required for further studies such as categorize parasitic infected children into different groups depending on the times of exposure to the parasitic infection and the severity of that infection.

## Data availability

The quantitative data used to support the findings of this study are available from the corresponding author upon request.

## Fund

Self-funded

## Contributions

Azza Sajid Jabbr: Supervision., Conceptualization, Methodology, Writing - Review & Editing; Suha Haithem Mohamme: Writing - Original Draft Software, Formal analysis; Nawal Khalil Ibrahim: Investigation,Data Curation and Visualization.

## Declaration of competing interest

The authors declare that there are no conflicts of interest.

## References

[1] Bruschi F., Araujo M.I., Harnett W., Pinelli E. (2013). Allergy and parasites. Journal of Parasitology Research.

[2] Sitcharungsi R., Sirivichayakul C. (2013). Allergic diseases and helminth infections. Pathog. Glob. Health.

[3] Cooper P.J. (2009). Interactions between helminth parasites and allergy. Allergy.

[4] Culver E.L. (2017). Increases in IgE, eosinophils, and mast cells can be used in diagnosis and to predict relapse of IgG4-related disease. Clin. Gastroenterol. Hepatol..

[5] Bethony J. (2006). Soil-transmitted helminth infections: ascariasis, trichuriasis, and hookworm. Lancet.

[6] Hadush A., Pal M. (2016). Ascariasis: public health importance and its status in Ethiopia. Air Water Borne Dis..

[7] Oliveira F.M.S. (2019). Comorbidity associated to ascaris suum infection during pulmonary fibrosis exacerbates chronic lung and liver inflammation and dysfunction but not affect the parasite cycle in mice. PLoS Neglected Trop. Dis..

[8] Cruz A.A., Cooper P.J., Figueiredo C.A., Alcantara-Neves N.M., Rodrigues L.C., Barreto M.L. (2017). Clinical reviews in allergy and immunology Global issues in allergy and immunology: parasitic infections and allergy. J. Allergy Clin. Immunol..

[9] Taghipour A., Rostami A., Sepidarkish M., Ghaffarifar F. (2020). Is Ascaris lumbricoides a risk factor for development of asthma? A systematic review and meta-analysis. Microb. Pathog..

[10] Tepper R.S. (2012). Asthma outcomes: pulmonary physiology. J. Allergy Clin. Immunol..

[11] Murray C., Foden P., Lowe L., Durrington H., Custovic A., Simpson A. (2017). Diagnosis of asthma in symptomatic children based on measures of lung function: an analysis of data from a population-based birth cohort study. Lancet Child Adolesc. Heal..

[12] Johnson J.D., Theurer W.M. (Mar. 2014). A stepwise approach to the interpretation of pulmonary function tests. Am. Fam. Physician.

[13] Jabbar A.S., Mohammed R.N. (2020). Impact of paints exposure on pulmonary function tests of male workers in basrah city,south of Iraq. Int. J. Pharmacol. Res..

[14] Ansotegui I.J. (Feb. 2020). IgE allergy diagnostics and other relevant tests in allergy, a World Allergy Organization position paper. World Allergy Organ. J..

[15] Hooshyar H., Rostamkhani P., Arbabi M., Delavari M. (2019). Giardia lamblia infection: review of current diagnostic strategies. Gastroenterology and Hepatology from Bed to Bench.

[16] Calogero C., Fenu G., Lombardi E. (2018). Measuring airway obstruction in severe asthma in children. Frontiers in Pediatrics.

[17] Nagarchi K., Ahmed S., Saheb S.H. (2015). Study of pulmonary function test in asthma patients. J. Pharmaceut. Sci. Res..

[18] Bacharier L.B., Strunk R.C., Mauger D., White D., Lemanske R.F., Sorkness C.A. (2004). Classifying asthma severity in children: mismatch between symptoms, medication use, and lung function. Am. J. Respir. Crit. Care Med..

[19] Nagarchi K., Ahmed S., Saheb S.H. (2015). Study of pulmonary function test in asthma patients. J. Pharmaceut. Sci. Res..

[20] Caraballo L., ResponAse Aah A.I. the A., Symptoms A., Cevedo N., Buendía E. (2015). Human ascariasis increases the allergic response and allergic symptoms. Curr. Trop. Med. Reports.

[21] Cooper P.J. (2009). Interactions between helminth parasites and allergy. Curr. Opin. Allergy Clin. Immunol..

[22] Weiss S.T. (2000). Parasites and asthma/allergy: what is the relationship?. J. Allergy Clin. Immunol..

[23] Saiphoklang N., Chomchoey C. (2020). Eosinophilia and parasitic infestations in patients with chronic obstructive pulmonary disease. Sci. Rep..

[24] Hoerauf A., Satoguina J., Saeftel M., Specht S. (Oct. 2005). Immunomodulation by filarial nematodes. Parasite Immunol..

[25] Santos A.B.R. (2008). Cross-reactive IgE antibody responses to tropomyosins from Ascaris lumbricoides and cockroach. J. Allergy Clin. Immunol..

[26] Sousa-Santos A.C.A.F. (2020). Parasite infections, allergy and asthma: a role for tropomyosin in promoting type 2 immune responses. Int. Arch. Allergy Immunol..

[27] Cooper P.J., Chico M.E., Bland M., Griffin G.E., Nutman T.B. (2003). Allergic symptoms, atopy, and geohelminth infections in a rural area of Ecuador. Am. J. Respir. Crit. Care Med..

[28] Dagoye D. (May 2003). Wheezing, allergy, and parasite infection in children in urban and rural Ethiopia. Am. J. Respir. Crit. Care Med..

[29] Anthony R.M., Rutitzky L.I., Urban J.F., Stadecker M.J., Gause W.C. (2007). Protective immune mechanisms in helminth infection. Nat. Rev. Immunol..

[30] Weatherhead J.E. (2020). Host immunity and inflammation to pulmonary helminth infections. Front. Immunol..

